# Eribulin in pretreated metastatic breast cancer patients: results of the TROTTER trial—a multicenter retrospective study of eribulin in real life

**DOI:** 10.1186/s40064-016-1700-0

**Published:** 2016-01-21

**Authors:** Ornella Garrone, Filippo Montemurro, Chiara Saggia, Nicla La Verde, Anna Maria Vandone, Mario Airoldi, Enrico De Conciliis, Michela Donadio, Francesco Lucio, Maria Antonia Polimeni, Maria Vittoria Oletti, Alice Giacobino, Marco Carlo Merlano

**Affiliations:** Medical Oncology, A.O. Ospedale di Insegnamento S. Croce e Carle, V. M. Coppino, 26, 12100 Cuneo, Italy; Investigative Clinical Oncology, Fondazione del Piemonte per l’Oncologia, Candiolo Cancer Institute (IRCCS), Strada Provinciale 142, 10060 Candiolo, Turin, Italy; Medical Oncology, A.O.U. Maggiore della Carità, C. G. Mazzini, 28, 28100 Novara, Italy; Department of Oncology, A.O. Fatebenefratelli & Oftalmico, C. di Porta Nuova, 25, 20121 Milan, Italy; Department of Medical Oncology 2, A.O.U. Città della Salute e della Scienza, C. Bramante, 88, 10126 Turin, Italy; Medical Oncology, Ospedale Cardinal Massaia, C. D. Alighieri, 201, 14100 Asti, Italy; Breast Unit, A.O.U. Città della Salute e della Scienza, C. Bramante, 88, 10126 Turin, Italy; Radiotherapy Oncology, A.O. Ospedale di Insegnamento S. Croce e Carle, V. M. Coppino, 26, 12100 Cuneo, Italy; Medical Oncology, Ospedale Ivrea, Piazza Credenza, 2, 10015 Ivrea, Italy; Medical Oncology, Ospedale S. Spirito, V. G. Giolitti, 2, 15033 Casale Monferrato, Italy; Department of Oncology, Ospedale degli Infermi, V. Ponderanesi, 2, 13875 Ponderano, Italy

**Keywords:** Metastatic breast cancer, Eribulin, Real life, Toxicity

## Abstract

This retrospective multicenter analysis was aimed to evaluate clinical activity and tolerability of eribulin in pretreated metastatic breast cancer patients in clinical practice. Patients treated with eribulin from January 2012 to July 2013 were enrolled in the observational study from 10 italian hospitals. Tumor and toxicity evaluation were performed according to Agenzia Italiana Farmaco. One-hundred and thirteen patients were included in the study. Median age 62 years old. 71.7 % of the patients had visceral involvement and the majority had a burden of disease involving two or more organs with a median number of 2 (1–6). The median number of previous chemotherapy regimens for advanced disease was 3 (1–10). Median number of eribulin cycles was 4 (1–27). Overall response rate was 24 % (95 % CI 16.0–31.8). Clinical benefit rate, was 35.4 % (95 % CI 26.6–44.2). At a median follow-up of 29.6 months (8.3–41.9) the median progression free survival was 3.3 months (0.6–26.7; 95 % CI 2.4–4.2), and the median overall survival 11.6 months (0.6–33.3; 95 % CI 8.7–14.5). No correlation was recorded between subtypes in terms of ORR and CBR. Toxicity was manageable. Main common grade 3–4 toxicities were neutropenia (19.4 %), febrile neutropenia (0.9 %), asthenia (3.5 %), abnormal liver function test (1.8 %), stomatitis (0.9 %). Our results confirm that treatment with eribulin is feasible and safe in real-world patients.

## Background

Metastatic breast cancer (MBC) remains an incurable disease despite considerable progress and new treatment options. The goals of therapy are disease stabilization or reduction in the total disease burden, extension of life expectancy and preservation of quality of life (Goldhirsch et al. 2013). Treatment strategies for patients with MBC should consider many factors, such as BC subtype (i.e. luminal or basal, HER2 enriched BC), the event-free interval, prior treatments, patient tolerability and preference.

Although endocrine manipulation and anti-HER2 agents represent pivotal “targeted” therapeutic choices for patients with endocrine responsive and HER2-positive MBC, respectively, cytotoxic chemotherapy remains a mainstay of treatment (Cardoso et al. 2014). In fact, chemotherapy is indicated in hormone-receptor positive breast cancer that is refractory to endocrine therapy, is a companion for anti-HER2 agents and as is the only registered treatment for so called “triple-negative” breast cancers, which lack hormone-receptor and HER2 expression. When disease progresses, MBC patients may derive sustained benefits from the administration of several lines of chemotherapy. Therefore, efforts are still concentrated on increasing the list of the available chemotherapy agents, in order to have more effective and well-tolerated options that could prolong the life expectancy of MBC patients.

Anthracyclines and taxanes are both standard treatment in the adjuvant setting, therefore, the majority of MBC patients have already been exposed to such agents.

There are several additional agents approved for the treatment of advanced disease such as capecitabine, vinca-alkaloid, gemcitabine, liposomal anthracycline and nanoparticle albumin-bound paclitaxel. One recent and notable addition to this repertoire is Eribulin mesylate.

Eribulin mesylate is a synthetic analogue of halichondrin B, a natural large polyether macrolide found in marine sponge Halicondria okadai with a distinct mechanism of inhibition of microtubule dynamics which differs from other types of tubulin-targeting drugs such as vinca alkaloids and taxanes. Eribulin suppresses the growth phase of microtubules, without affecting the shortening phase, and sequesters tubulin into non-productive aggregates (Jordan et al. 2005). Therefore it prevents the formation of mitotic spindle, which results in irreversible mitotic block, cell cycle arrest in the G2-M phase and apoptosis (Jordan et al. 2005; Okouneva et al. 2008; Dabydeen et al. 2006; Kuznetsov et al. 2004).

Preclinical studies showed activity against breast, colon, prostate and melanoma cell lines (Towle et al. 2001). In phase II trials it demonstrated activity both in pretreated and in previously untreated patients. ORR has ranged from 9.3 to 21.3 % in pretreated patients and from 28.6 % (Her2 negative) to 71.2 % (HER2 positive) in previously untreated ones (Vahdat et al. 2009; Aogi et al. 2012; Cortes et al. 2010; McIntyre et al. 2014; Wilks et al. 2014).

The EMBRACE trial, a randomized phase III study comparing eribulin with the treating physician’s choice (TPC) in heavily pretreated breast cancer patients, found that patients treated with eribulin experienced a significant survival advantage, with manageable toxicity (Cortes et al. 2011). The most common side effects were neutropenia, fatigue and peripheral neuropathy. The European Medicines Agency (EMA) approved eribulin in the treatment of patients with MBC exposed to a minimum of two previous chemotherapy regimens including anthracyclines and taxanes in the adjuvant or metastatic setting. The population of the EMBRACE study was similar to that seen in daily clinical practice, leading to approval of the drug for the treatment of MBC patients after second line.

To evaluate the role of eribulin in daily clinical practice the authors performed a retrospective analysis to describe the use of the drug and its activity and safety.

## Methods

### Ethics statement

The study was approved by local ethical committees. Being a retrospective analysis of clinical outcomes, no specific written informed consent was required. Patients’ records were anonymized and de-identified prior to analysis.

### Patients

The authors reviewed retrospective data of all advanced breast cancer patients treated at 10 italian hospitals, with eribulin from January 2012 (availability of the drug in Italy) to July 2013.

Patients diagnosed with advanced breast cancer and candidate for treatment with eribulin according to EMA criteria were considered for the study. Patients treated at the approved dose of 1.23 mg/m^2^ (equivalent to 1.4 mg/m^2^ eribulin mesylate) infused over 2–5 min intravenously on days 1 and 8, every 3 weeks were accrued. Treatment was continued until disease progression, severe toxicity or patient refusal. Dose reduction or cycles delayed were described.

Treatment response was assessed by RECIST criteria every 2 cycles during the first 4 courses of treatment and subsequently every 3 cycles according to rules of the Agenzia Italiana del Farmaco (AIFA) registry. Toxicity was assessed every cycle according to National Cancer Institute Common Terminology Criteria for Adverse Events (NCI-CTCAE, version 4).

### Statistical analysis

The purpose of the study was to evaluate the clinical outcome of the treated patients in terms of progression free survival (PFS), objective response rate (ORR) and the correlation with biological features, safety and overall survival (OS). Response rate included complete response (CR) and partial response (PR), The ORR was reported with its 95 % confidence interval (95 % CI). Clinical benefit (CB) was defined as CR + PR + disease stabilization (SD) lasting at least 24 weeks. Disease control rate (DCR) was defined as CR + PR + SD.

PFS was the interval from the start of therapy with eribulin to the date of progression. Patients without progression were censored, progression free at the date of last follow-up. OS was calculated as the interval from the start of therapy with eribulin to the date of death or the date of last follow-up evaluation.

PFS and OS were calculated by Kaplan–Meier method.

## Results

From January 2012 to July 2013, 113 advanced breast cancer patients were treated with eribulin in 10 Italian cancer centers. Patients were eligible if they had received at least 1 cycle of eribulin by the end of July 2013. Main patients’ characteristics are listed in Table 1. Median age at eribulin initiation was 62 years (range 33–80), median ECOG performance status (PS) 1 (range 0–2). Ten patients (8.8 %) had metastatic disease on presentation.

**Table 1:** Patient demographics and baseline characteristics

Characteristics	N 113	%
Age (median, range)	62 (33–80)	
ECOG PS (median, range)	1 (0–2)	
De novo metastatic disease	10	(8.8)
ER status		
Positive	87	(77.0)
Negative	26	(23.0)
PgR status		
Positive	72	(63.7)
Negative	38	(33.6)
Unk	3	(2.7)
Triple negative	22	(19.5)
HER2 status		
Positive	11	(9.7)
Negative	95	(84.1)
Unk	7	(6.2)
Neo/adjuvant chemotherapy	82	(72.5)
Adjuvant hormonotherapy	67	(59.3)
Adjuvant trastuzumab	4	(3.5)
Number of prior chemotherapy for advanced disease		
1	8	(7.1)
2	26	(23.0)
3	34	(30.1)
4	20	(17.7)
5	14	(12.4)
≥6	11	(9.7)
Median, range	3 (1–10)	
Prior hormonotherapy for advanced disease	79	(69.9)
Median, range	2 (1–5)	
Number of organs involved		
1	17	(15.0)
2	43	(38.1)
3	33	(29.2)
≥4	20	(17.7)
Median, range	2 (1–5)	
Most common metastatic sites		
Bone	81	(71.7)
Liver	60	(53.1)
Lymph nodes	48	(42.5)
Lung	36	(31.8)
Skin	19	(16.8)
CNS	13	(11.5)
Rechallenge with anthracyclines and/or taxanes	64	(56.6)

*ER* estrogen receptor, *PgR* progesterone receptor, *HER2* human epidermal growth factor 2, *CNS* central nervous system

The most common metastatic sites were bone, liver, lymph-nodes and lung; overall 81 patients (71.7 %) had visceral involvement. The majority of patients had a burden of disease involving two or more organs with a median number of 2 (range 1–6) sites. Regarding treatment for metastatic disease 23 % of the patients had been treated with 2 previous chemotherapy regimens, 70 % were exposed to more than 2 previous lines and 8 patients (7 %) received eribulin as second line treatment. The median number of previous chemotherapy regimens for advanced disease was 3 (range 1–10). However, considering only patients treated in 2013, the previous chemotherapy regimens were 4. All but 14 patients (12.4 %) had received anthracyclines and taxanes as adjuvant or advanced therapy. Furthermore 43.4 % (49 out of 113) of cases in the current cohort had been rechallenged with taxanes during their disease course before receiving eribulin. Ninety-nine patients (87.6 %) received previous capecitabine.

Overall 611 cycles of eribulin were administered and the patients were exposed to a median of 4 cycles (range 1–27). Thirty-three patients (28.3 %) needed dose reduction and overall 101 cycles (16.5 %) were administered at a lower dose. Eribulin treatment was delayed in 33 cycles (5.4 %) in 23 patients (20.3 %).

Haematological toxicity was reported in Table 2. Grade 4 neutropenia occurred in 4 patients (3.5 %). One patient experienced grade 3 febrile neutropenia. Intriguingly 3 cases of grade 1 thrombocytosis were described.

**Table 2:** Haematological toxicity

	All grades (%)	Grade 3 (%)	Grade 4 (%)
Leucopenia	19 (16.8)	5 (4.4)	–
Neutropenia	41 (36.3)	18 (15.9)	4 (3.5)
Febrile neutropenia	1 (0.9)	1 (0.9)	–
Anaemia	32 (28.3)	3 (2.6)	–
Thrombocytopenia	8 (7.1)	1 (0.9)	–
Thrombocytosis	3 (2.6)	–	–

Regarding non-haematological toxicity (Table 3) 49 patients (43.4 %) experienced asthenia, being grade 3 in five patients (4.4 %). The only grade 4 toxicity was peripheral neuropathy reported by one patient (0.9 %).

**Table 3:** Non-hematological toxicity

	All Grades (%)	Grade 3 (%)	Grade 4 (%)
Astenia/fatigue	49 (43.4)	5 (4.4)	–
Peripheral neuropathy	14 (12.4)	–	1 (0.9)
Arthralgia/myalgia	9 (7.9)	–	–
Hypertransaminasemia	8 (7.1)	1 (0.9)	–
Abnormal liver function test	18 (15.9)	2 (1.8)	–
Stomatitis	5 (4.4)	1 (0.9)	–
Nausea/vomiting	9 (7.9)	–	–
Alopecia	19 (16.8)	–	–
Diarrhea	6 (5.3)	–	–
Constipation	3 (2.6)	–	–
Cough	2 (1.8)	–	–
Dyspnea	4 (3.5)	1 (1.09)	–

Mucositis was uncommon. Five patients reported grade 1–2 and 1 patient grade 3. Increase in transaminases was described in eight patients, and 1 experienced grade 3. In total, eighteen patients had liver toxicity.

Twenty-five patients (22.1 %) were aged 70 years or more. Toxicity in this subset was similar as in the whole population: grade 3 and 4 neutropenia in 4 (16.0 %) and 1 patient (4.0 %) respectively and grade 3 fatigue in 2 patients (8.0 %).

One hundred six patients were evaluated for response. Nine patients received only 1 cycle of eribulin: early progression in 2 and deterioration of clinical condition or toxicity in 7. These patients were considered not evaluable for response. No patient achieved a complete response. Twenty-seven partial responses were recorded for an overall response rate of 24 % (95 % CI 16.0–31.8). Stable disease was observed in 29 patients (25.7 %) and a clinical benefit rate, was obtained by 40 patients (35.4 %; 95 % CI 26.6–44.2). Disease control rate was recorded in 56 patients (49.5 %) (Table 4).

**Table 4:** Treatment efficacy

	N 113	(%)
Total number of chemotherapy cycles	611	
Median number of chemotherapy cycles (range)	4	(1–27)
Dose reduction (patients)	32	(28.3)
Dose reduction (cycles)	101	(16.5)
Cycle delay (patients)	23	(3.7)
Cycle delay (no)	33	(5.4)
Tumor response		
Complete response	–	
Partial response	27	(24.0)
95 % CI	(16.0–31.8)	
Stable disease	29	(25.7)
Progressive disease	51	(45.1)
Not evaluable	7	(6.2)
Disease control rate	56	(49.5)
Clinical benefit	40	(35.4)
95 % CI	26.6–44.2	
Median PFS (months, range)	3.3 (0.6–26.7)	
95 % CI	2.4–4.2	
Median OS (months, range)	11.6 (0.6–33.3)	
95 % CI	8.7–14.5	

*CI* confidence interval

In our retrospective cohort, 49 patients (43.4 %) had been rechallenged with taxanes. In these subgroup ORR was significantly higher 38.8 (19 out of 49) than the response in patients not rechallenged 14.1 % (9 out of 64) (p = 0.005). No significant difference was recorded in CBR 42.8 (21 out of 49) versus 29.7 % (19 out of 64).

No difference in PR and CB was observed in our cohort of unselected patients according to prognostic factors, site of disease, disease involvement and prior chemotherapy lines (Tables 5, 6).

**Table 5:** Response by tumor subtypes and treatment line

Characteristics	Partial response N (%)	*P*
Triple negative		
Yes	8 (30.7)	n.s.
Not	19 (21.8)	
ER/PgR status		
ER and/or PgR positive 88	18 (20.5)	n.s.
ER and/or PgR negative 30	9 (30)	
HER2		
Overexpressed/amplified	4 (33.3)	n.s.
Not overexpressed/amplified	17 (18.2)	
Dominant disease site		
Visceral	22 (27.1)	n.s.
Non visceral	5 (15.6)	
Number of metastatic disease		
1	3 (16.7)	n.s.
≥2	24 (25.3)	
Number of prior chemotherapy for advanced disease		
2	8 (30.7)	n.s.
≥3	19 (21.8)

**Table 6:** Clinical benefit by tumor subtypes and treatment line

Characteristics	Clinical benefit N (%)	*P*
Triple negative		
Yes	6 (23.1)	n.s.
Not	30 (34.5)	
ER/PgR status		
ER and/or PgR positive	28 (33.3)	n.s.
ER and/or PgR negative	12 (41.4)	
HER2		
Overexpressed/amplified	6 (46.1)	n.s.
Not overexpressed/amplified	32 (34.8)	
Dominant disease site		
Visceral	34 (40)	0.07
Non visceral	6 (21.4)	
Metastatic disease site		
1	5 (27.8)	n.s.
≥2	35 (36.8)	
Number of prior chemotherapy for advanced disease		
2	12 (35.3)	n.s.
≥3	28 (35.4)	

**Fig. 1:**
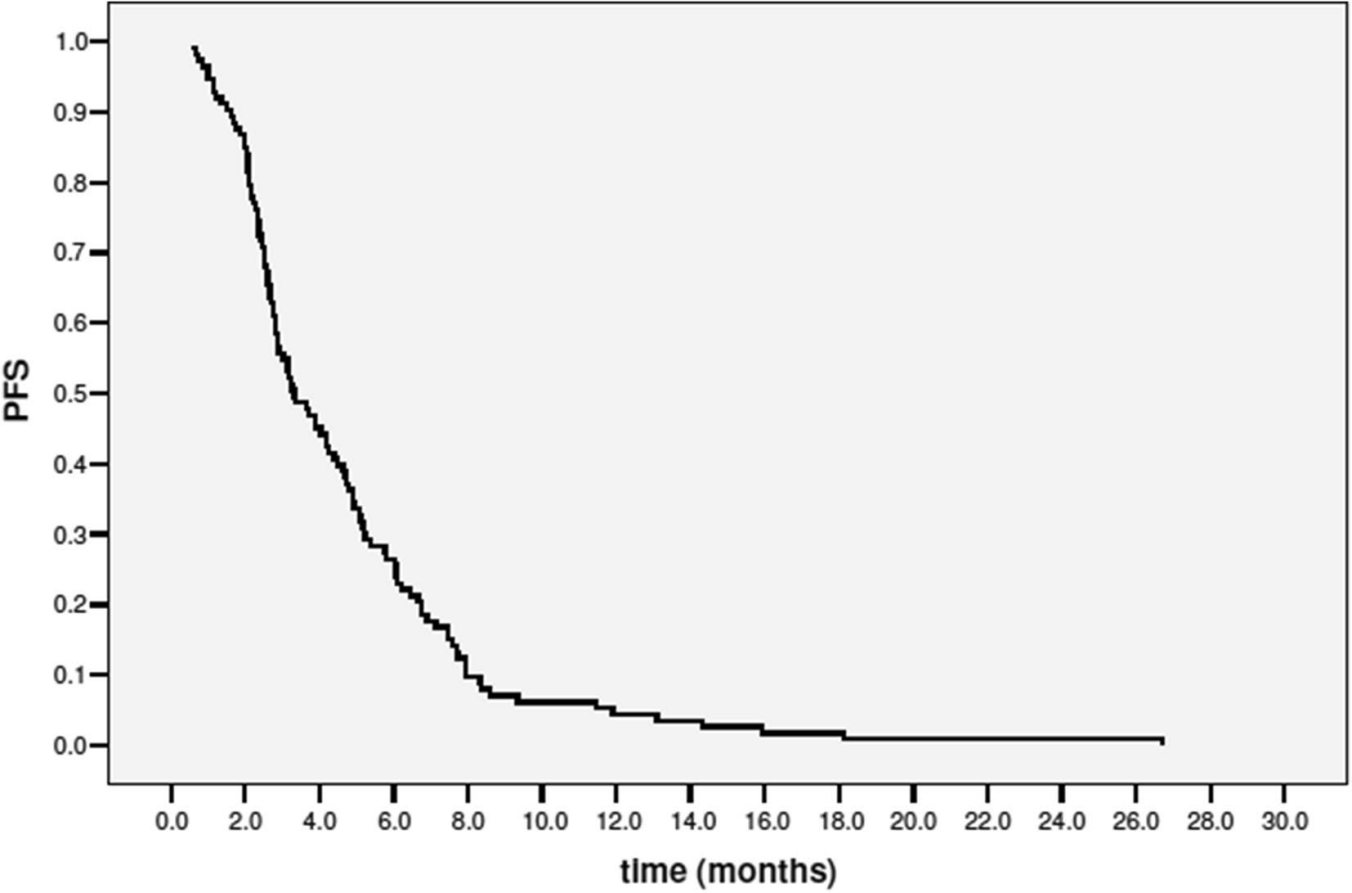
Progression free survival

**Fig. 2:**
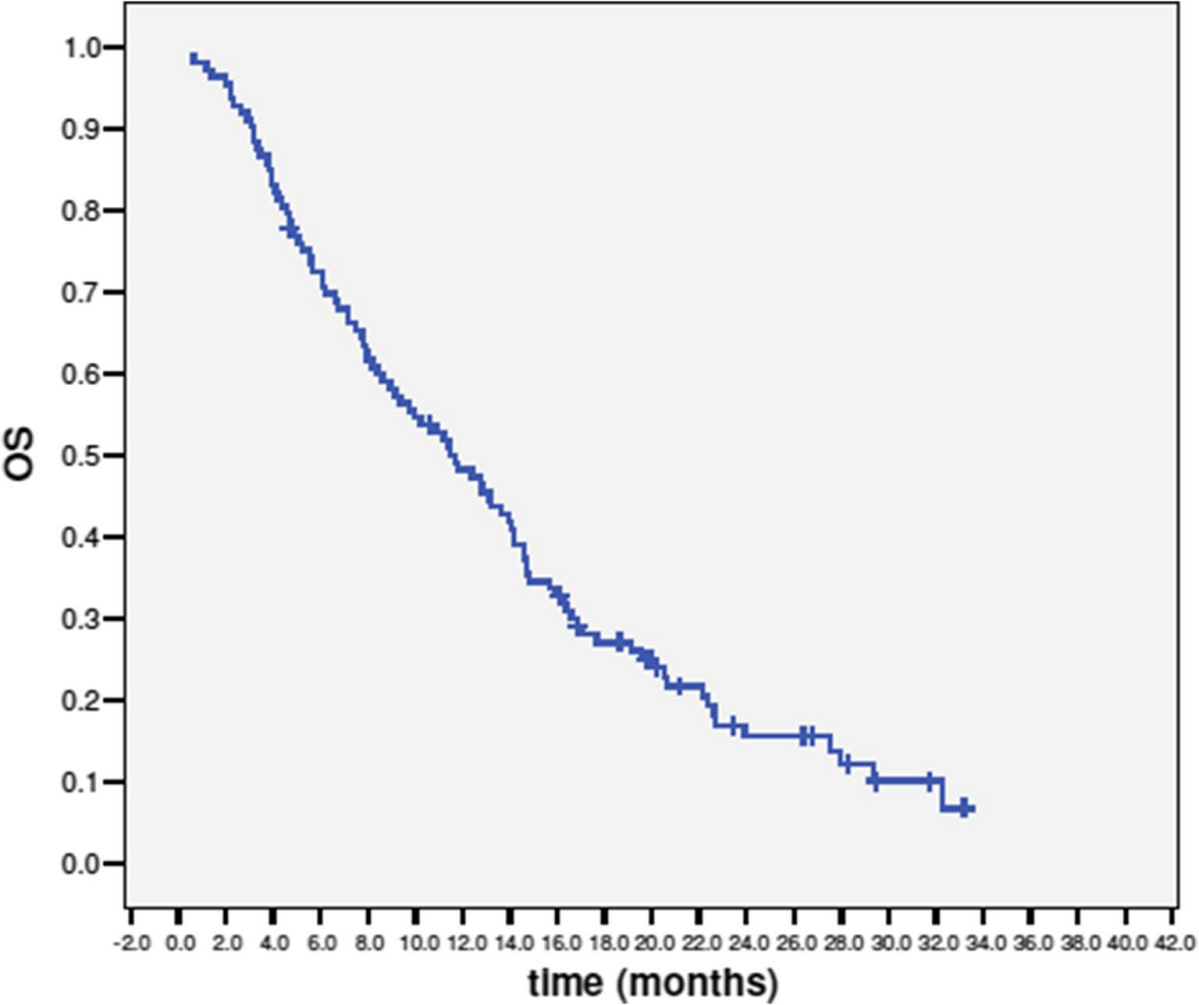
Overall survival

At the time of the present analysis 17 patients (15.0 %) were still alive and 1 was still in response. At a median follow-up of 29.6 months (range 8.3–41.9 months) the median progression free survival was 3.3 months (range 0.6–26.7 months; 95 % CI 2.4–4.2) (Fig. 1), and the median overall survival 11.6 months (range 0.6–33.3 months; 95 % CI 8.7–14.5) (Fig. 2).

A further analysis addressed to patients who received eribulin as second, third or fourth line of therapy, identified sixty-eight patients (60.2 %). In this group partial responses were 15 (25.0 %), clinical benefit was recorded in 24 patients (40 %) and median progression free survival was 4.1 months (range 0.7–26.7; 95 % CI 2.7–5.5).

Seventy-six patients (67.2 %) had further treatments after progression: 22 patients (19.5 %) received endocrine therapy, 54 patients (47.8 %) were exposed to chemotherapy and the remaining 37 patients (32.7 %) received best supportive care.

## Discussion

In this retrospective analysis, we studied patterns of use and clinical activity of eribulin in patients treated with this drug as a standard of care after it became available in our Country. Because of the strict selection criteria applied in clinical trials, which may select a population with favorable features, we sought to assess the reproducibility of the practice-changing results of the EMBRACE trial in a “real life” scenario.

Our main findings suggest that eribulin in this unselected patient population achieved similar results as reported in the EMBRACE study in terms of both activity and toxicity.

As all retrospective analyses, this study has limitations that must be acknowledged before commenting on the potential implications of our findings. Because we focused on safety and activity, lack of a prospective protocol mandating data collection and timing of assessment may be seen as a major limitation. However, since the approval or eribulin for clinical use in Italy, prescription is possible only through the Agenzia Italiana del Farmaco (AIFA) registry. This web-based National registry regulates prescription appropriateness and reimbursement to oncology centers. The registry requires mandatory input of treatment compliance, serious adverse events, drug dosing before each prescription, and tumor response, which needs to be assessed at specific time-points. Therefore, although we did not extract data from the Registry, because it is not allowed, we can be confident that side effects collection and disease assessments were consistent across centers in this patient cohort. For this reason, we believe that our study has findings that may be of potential interest for the practical use of eribulin as a standard of care.

First, about 20 % of our patients did not match the EMA indications. This may reflect the initial enthusiasm that a new drug induces in the medical community, when its approval is supported by convincing data. Over the time we assisted to a more appropriate prescription of the drug. In our cohort the previous median chemotherapy lines was 4 in 2012 and 3 in 2013.

Second, the toxicity observed in this unselected population was acceptable and in line with that reported in the EMBRACE. This aspect deserves consideration because median age in our cohort was older than the median age in the pivotal study (62 vs. 55 respectively). Moreover we did not observe differences in toxicity between young and elderly patients, considering that 22 % of patients were 70 years or older. The same observation was also reported by Gamucci et al. (2014) in a retrospective cohort of advanced breast cancer patients and by Muss et al. (2015) in a pooled analysis of two phase II trials and one phase III trial. Taken together these features confirm the favorable toxicity profile of eribulin in a “real life” scenario. The relative low rate of neuropathy in our series may be mainly due to the fact that 21 patients (18.6 %) were exposed to no more than 2 cycles of chemotherapy.

We observed toxicities not reported in the EMBRACE, such as liver toxicity and thrombocytosis. Increase in transaminases is quite common using eribulin and it has been described in other reports dealing with real life patients (Gamucci et al. 2014; Poletti et al. 2014; Ramaswami et al. 2014). On the contrary, to our knowledge no other experiences reported on thrombocytosis; we have no clear explanation for this effect. It could represent a platelet rebound after a transient thrombocytopenia, but this is speculative.

Third, our data confirm that eribulin maintains its favorable profile in terms of clinical activity (ORR, CBR, PFS and OS) in daily clinical practice in highly pretreated patients. We recorded an overall response rate of 24 %, a clinical benefit of 35.4 %, a median progression free survival of 3.3 months and a median overall survival of 11.6 months. All these results are comparable to the data within the EMBRACE.

We did not observe any significant difference of activity among biological subgroups and clinical characteristics but we have to consider the relative small number of patients. Indeed we observed activity in all metastatic sites, in triple negative patients as well as in hormone receptor or HER2 positive patients.

No significant difference was observed in terms of objective response among patients exposed to eribulin as third or fourth line and patients treated in subsequent lines (25 vs. 25.7 %). A slight, not significant, increase in clinical benefit (40 vs. 35.4 %) and progression free survival (4.1 vs. 3.3 months) was observed in the cohort of patients treated in earlier lines. The lack of significant differences might be due to the relative small number of patients and deserves further confirmation. Gamucci et al. (2014) reported a significant difference in clinical benefit in patients exposed to eribulin as third line in comparison with more advanced lines.

In a small cohort of 25 patients, rechallenge with anthracyclines and/or taxanes was associated with a nonsignificant difference in ORR and with a significant difference in CBR favoring patients who had not been rechallenged. TTP was longer too in this subset of patients (Ramaswami et al. 2014).

In contrast with that reported by Ramaswami et al. (2014) in our report patients exposed to a rechallenge with taxanes obtained a greater benefit in ORR compared with patients not re-exposed to taxanes. We can speculate that the previous sensibility to taxanes could be predictive of eribulin sensitivity as they share the same site of action.

Overall 67.2 % (76 out of 113) of the patients progressing on eribulin received further treatments. Among the 54 patients treated with chemotherapy at progression, about 15 % were treated with more than one line of chemotherapy. This result highlights the good tolerability of the drug in heavily pretreated metastatic breast cancer.

## Conclusions

In summary, notwithstanding the limitations of the retrospective analysis, our results depict the outcome of an unselected patient population representative of the “real life”.

It is well known the difficulty to translate the results obtained in a clinical trial, with a new drug, to the daily clinical practice, due to the strict patients’ selection.

However, our data, together with similar retrospective analyses conducted in daily care conditions, are consistent with the results observed in the registrative study EMBRACE, and support the use of eribulin in heavily pretreated metastatic breast cancer patient.

## Abbreviations

MBC: metastatic breast cancer
BC: breast cancer
PFS: progression free survival
OS: overall survival
ORR: objective response rate
CR: complete response
PR: partial response
CB: clinical benefit
SD: stable disease
DCR: disease control rate
PS: performance status

## References

[CR1] Aogi K, Iwata H, Masuda N, Mukai H, Yoshida M, Rai Y (2012). A phase II study of eribulin in Japanese patients with heavily pretreated metastatic breast cancer. Ann Oncol.

[CR2] Cardoso F, Costa A, Norton L, Senkus E, AaproM André F (2014). ESO-ESMO 2^nd^ international consensus guidelines for advanced breast cancer (ABC2). Breast.

[CR3] Cortes J, Vahdat L, Blum JL, Twelves C, Campone M, Roché H (2010). Phase II study of the halichondrin B analog eribulin mesylate in patients with locally advanced or metastatic breast cancer previously treated with an anthracycline, a taxane, and capecitabine. JCO.

[CR4] Cortes J, O’Shaughnessy J, Loesh D, Blum JL, Vadhat LT, Petrakova K (2011). On behalf of the Embrace (Eisai Metastatic Breast Cancer Assessing Physician’s Choice Versus E7389) investigators. Lancet.

[CR5] Dabydeen DA, Burnett JC, Bai R, Verdier-Pinard P, Hickford SJ, Pettit GR (2006). Comparison of the activities of the truncated halichondrin B analog NSC 707389 (E7389) with those of the parent compound and a proposed binding site on tubulin. Mol Pharmacol.

[CR6] Gamucci T, Michelotti A, Pizzuti L, Mentuccia L, Landucci E, Sperduti I (2014). Eribulin mesylate in pretreated breast cancer patients: a multicenter retrospective observational study. J Cancer.

[CR7] Goldhirsch A, Winer EP, Coates AS, Gelber RD, Piccart-Gebhart M, Thürlimann B (2013). Personalizing the treatment of women with early breast cancer: highlights of the St Gallen International Expert Consensus on the Primary Therapy of Early Breast Cancer 2013. Ann Oncol.

[CR8] Jordan MA, Kamath K, Manna T, Okouneva T, Miller HP, Davis C (2005). The primary antimitotic mechanism of action of the synthetic halichondrin E7389 is suppression of microtubule growth. Mol Cancer Ther.

[CR9] Kuznetsov G, Towle MJ, Cheng H, Hawamura T, TenDyke K, Liu D (2004). Induction of morphological and biochemical apoptosis following prolonged mitotic blockage by halichondrin B macrocyclic ketone analog E7389. Cancer Res.

[CR10] McIntyre K, O’Shaughnessy J, Schwartzberg L, Glück S, Berrak E, Song JX (2014). Phase 2 study of eribulin mesylate as first-line therapy for locally recurrent or metastatic human epidermal growth factor receptor 2-negative breast cancer. Breast Cancer Res Treat.

[CR11] Muss H, Cortes J, Vahdat LT, Cardoso F, Twelves C, Wanders J (2015). Eribulin monotherapy in patients aged 70 years and older with metastatic breast cancer. Oncologist.

[CR12] Okouneva T, Azarenko O, Wilson L, Littlefield BA, Jordan MA (2008). Inhibition of centromere dynamics by eribulin (E7389) during mitotic metaphase. Mol Cancer Ther.

[CR13] Poletti P, Ghilardi V, Livraghi L, Milesi L, Rota E, Tondini C (2014). Eribulin mesylate in heavily pretreated metastatic breast cancer patients: current practice in an Italian community hospital. Future Oncol.

[CR14] Ramaswami R, O’Cathail SM, Brindley JH, Silcocks P, Mahmoud S, Palmieri C (2014). Activity of eribulin mesylate in heavily pretreated breast cancer granted access via cancer drugs fund. Future Oncol.

[CR15] Towle MJ, Salvato KA, Budrow J, Wels BF, Kuznetsov G, Aalfs LL (2001). In vitro and in vivo anticancer activities of synthetic macrocyclic ketone analogues of halichondrin B. Cancer Res.

[CR16] Vahdat LT, Pruitt B, Fabian CJ, Rivera RR, Smith DA, Tan-Chiu E (2009). Phase II study of eribulin mesylate, a halichondrin B analog, in patients with metastatic breast cancer previously treated with an anthracycline and a taxane. J Clin Oncol.

[CR17] Wilks S, Puhalla S, O’Shaughnessy J, Schwartzberg L, Berrak E, Song J (2014). Phase 2, multicenter, single-arm study of eribulin masylate with trastuzumab as first-line therapy for locally recurrent or metastatic HER2-positive breast cancer. Clin Breast Cancer.

